# Genetic variation in 9p21, dietary patterns, and insulin sensitivity

**DOI:** 10.3389/fgene.2022.988873

**Published:** 2022-10-14

**Authors:** Sara Mahdavi, David J.A. Jenkins, Ahmed El-Sohemy

**Affiliations:** ^1^ Department of Nutritional Sciences, Faculty of Medicine, University of Toronto, Toronto, ON, Canada; ^2^ Li Ka Shing Knowledge Institute, Risk Factor Modification Centre and Division of Endocrinology and Metabolism, St. Michael’s Hospital, St. Michael’s Health Centre, Toronto, ON, Canada

**Keywords:** nutrigenetics, personalized nutrition, nutrigenomics, 9p21, insulin sensitivity, precision nutrition, gene-diet interactions

## Abstract

**Background:** Single nucleotide polymorphisms in the 9p21 region have been associated with cardiovascular disease and to a lesser extent insulin sensitivity. Previous studies have focused on older populations, and few have examined the impact of gene-diet interactions. The objective of this study was to determine the interaction between dietary patterns and 9p21 genotypes on insulin sensitivity in young adults from different ethnic groups.

**Methods:** Subjects were 1,333 participants aged 20–29 years from the Toronto Nutrigenomics and Health Study (405 men and 928 women; 776 Caucasians and 557 East Asians). Fasting blood was collected to measure glucose, insulin, c-reactive protein and serum lipids, as well as to isolate DNA for genotyping subjects for five SNPs in 9p21 (rs10757274, rs10757278, rs1333049, rs2383206, and rs4977574). Insulin resistance (HOMA-IR) and beta-cell dysfunction (HOMA-Beta) were calculated from fasting insulin and glucose concentrations. The Toronto-modified Harvard 196-item semi-quantitative food frequency questionnaire was used to measure dietary intake over 1 month and principal components analysis was used to identify three dietary patterns (Prudent, Western and Eastern). ANOVA and ANCOVA were used to examine gene-diet interactions on markers of insulin sensitivity.

**Results:** Significant gene-diet interactions on insulin sensitivity using HOMA-IR were observed with all five SNPs, which remained significant after adjusting for covariates (*p* < 0.05). Among those who were homozygous for the 9p21 risk allele (rs1333049), fasting insulin was 40% higher in those who were consuming a low-prudent diet compared to those consuming a high-prudent diet (*p* < 0.05). No differences were observed between those following a low *versus* high-prudent diet among those who did not carry a 9p21 risk allele. Similar findings were observed with HOMA-Beta, however, the association was only significant for rs10757274 (*p* = 0.04).

**Conclusion:** Our findings suggest that a prudent dietary pattern may protect against the effects of 9p21 risk genotypes on insulin sensitivity.

## Introduction

Single nucleotide polymorphisms (SNPs) in the 9p21 region increase risk of CVD events, with a population attributable risk of 20–30% for myocardial infarction (Helgadottir et al., 2007). Two common SNPs at the 9p21 locus were largely responsible for CAD risk stratification in a recent cohort of genetically diverse adults (Tcheandjieu et al., 2022). SNPs in 9p21 have also been associated with type 2 diabetes (DM2) and IR (Cugino et al., 2012), suggesting a possible common genetic predisposition. The mechanisms by which 9p21 risk variants impact the pathogenesis of CVD and DM2 remain unknown since the region does not contain annotated genes (Helgadottir et al., 2007; McPherson et al., 2007). However, alteration in response to inflammatory signaling in human vascular endothelial cells has been observed in those with risk alleles (Harismendy et al., 2011; Xia et al., 2021). More recently a non-protein-coding DNA, ANRIL in the 9p21 region and adjacent CDKN2A/B tumor suppressors has been associated with atherogenesis (Holdt and Teupser, 2018; Razeghian-Jahromi et al., 2022). Alterations in IncRNA such as ANRIL and CDKN2A/B have been associated with poor glycemic control, insulin resistance, and inflammation, however the exact molecular mechanisms are yet to be uncovered (Sathishkumar et al., 2018; Xia et al., 2021).

Insulin resistance (IR) and glucose intolerance in young adults are risk factors for cardiovascular disease (CVD) in middle and late adulthood, even in those without diabetes (Gast et al., 2012). IR and endothelial dysfunction (ED) have some common biological mechanisms that stem from similar lifestyle and genetic factors (Kim et al., 2006). In addition to traditional pathways, IR has also been linked to atherosclerosis acting through pro-inflammatory pathways on vascular and immune cells (Bertoni et al., 2007; Montecucco et al., 2008). Genetic predisposition as well as dietary intake can modify insulin resistance, however, in most research and clinical settings, these factors are not considered together.

One of the most important environmental exposures that modify risk of IR and CVD, is dietary intake (Hu et al., 2000; Jenkins et al., 2007; Brunner et al., 2008; Salas-Salvadó et al., 2008; Mena et al., 2009; Urpi-Sarda et al., 2012). Some dietary patterns such as the Mediterranean diet, Portfolio diet and prudent dietary patterns, have been shown to significantly reduce CVD risk independent of other lifestyle factors (Hu et al., 2000; Jenkins et al., 2007; Brunner et al., 2008; Salas-Salvadó et al., 2008; Mena et al., 2009; Urpi-Sarda et al., 2012). However, few studies have reported on the interaction between diet and genetic risks, such as those in 9p21 (Do et al., 2011; Hindy et al., 2014). In one study that analyzed gene-diet interactions in several distinct populations, some food groups were found to modify CVD risk related to 9p21 genotype (Do et al., 2011). Of note, the effect of diet on CVD markers was only observed in risk allele carriers, but not others (Do et al., 2011). However, the study was conducted in older adults with CVD events and matched controls. To date, no studies have examined 9p21 gene-diet interactions in younger adults. Given the long lag times between exposure to risks and CVD outcomes, emphasis on preventive approaches to modify CVD at an earlier age may be more advantageous than later in life (Pencina et al., 2009; Batsis and Lopez-Jimenez, 2010; Balagopal et al., 2011). Since CVD risks take several decades to develop into CVD events, studying gene-diet interactions in younger adults might be more advantageous in identifying biological mechanisms of CVD risk with fewer confounders. We previously observed an association between 9p21 genotype and fasting insulin (Mahdavi et al., 2018). The current study aims to extend those findings to determine whether 9p21 risk variants interact with dietary patterns to impact insulin resistance in a population of young adults from different ethnic groups.

## Methods

### Study population

Participants were from the Toronto Nutrigenomics and Health Study (TNHS), which has been described elsewhere (García-Bailo et al., 2012). In brief, the TNHS is a cross-sectional study that aims to explore the link between diet, genes and biomarkers of chronic disease and was approved by the University of Toronto Research Ethics Board. Study participants were aged 20–29 years from various ethno-cultural backgrounds and were recruited from the University of Toronto campus between 2004 and 2010. Anthropometric measurements including height, weight, waist circumference, and blood pressure were recorded according to standard procedures (García-Bailo et al., 2012). Subjects provided a fasting blood sample for DNA isolation and plasma was separated for measuring biomarkers of CVD risk including blood lipids, inflammatory markers, glucose and insulin. The current study included 1,333 subjects from the two largest ethnic groups (Caucasian and East Asian) who had complete dietary and genetic data available for analyses.

### Clinical characteristics

Anthropometric measurements including height, weight, waist circumference, and blood pressure were measured using standard procedures described elsewhere (García-Bailo et al., 2012). Subjects also answered a general health and lifestyle questionnaire, indicating any medication use, including contraceptive use (Josse et al., 2012). Physical activity was assessed using a validated physical activity questionnaire and expressed as metabolic equivalent (MET) hours per week (Ainsworth et al., 1993). Subjects provided a 12-h overnight fasting blood sample for DNA isolation and plasma was separated for measuring biomarkers of interest as glycemic measures, lipids, inflammatory markers and insulin previously described (Cahill et al., 2009). Homeostatic measure of insulin resistance (HOMA-IR) and Beta cell function (HOMA-Beta) were calculated using validated mathematical formulae; HOMA-IR=(insulin X glucose)/22.5 and HOMA-Beta=(20 X insulin)/(glucose - 3.5) (Matthews et al., 1985).

### Dietary analyses

Dietary intake was assessed using the Toronto-modified Harvard 196-item semi-quantitative food frequency questionnaire (FFQ) (Cahill et al., 2009). In brief, each subject was given instructions on how to complete the FFQ by using visual aids of portion sizes to improve the measurement of self-reported food intake. This estimated the quantity and frequency of the food items and supplements consumed over 1 month. Subject responses to the individual foods were converted into daily number of servings for each item (Brenner et al., 2011) and supplement use. Since dietary patterns are better at predicting overall disease risk than individual nutrients or foods (Hu, 2002), dietary patterns were assessed using principal components analysis (PCA). Individual food items in the FFQ were used as the basis of the PCA, and to better describe the pattern structure of clusters, explained in detail elsewhere (Brenner et al., 2011).

The formation of dietary patterns for this cohort have been described previously (Brenner et al., 2011). Three main dietary patterns were identified as “Prudent” “Western,” and “Eastern” patterns. Prudent pattern consisted of fruit, vegetables, nuts, lentils, beans, whole grains, and water. Eastern pattern consisted of vegetables, seafood, rice, and organ meat. Western pattern consisted of processed foods, high-saturated fat, salty and sugary foods, refined grain products, and sugary beverages. Each of the three dietary patterns had a composite score indicating habitual dietary adherence of the subjects to the diets over a 1-month period. These scores where converted into three categories for each pattern where “low” indicated a score in the 25th or lower percentile of group score distribution, “medium” within 26th-74th percentile, and “high” indicating 75th or higher percentile for each of the three dietary patterns.

### Genotyping

DNA was analyzed for the following five SNPs in 9p21: rs1333049, rs10757278, rs2383206, rs10757274, and rs4977574 that have been shown most consistently to be associated with CVD risk (McPherson, 2010; Roberts, 2015). Genotyping was completed at the Clinical Genomics Centre in the Princess Margaret Hospital, University Health Network, using the iPLEX Gold assay with mass spectrometry-based detection (Sequenom MassARRAY platform; Sequenom Inc.). Since the presence of two risk alleles in the 9p21 region have been associated with the highest risk of CVD (McPherson et al., 2007) subjects were grouped in two groups according to having two copies of the risk alleles *versus* having one or more non-risk alleles. For examples, rs1333049 non-risk allele is G hence low CVD risk carriers were those with either a GG or CG genotype. High CVD risk group was defined as those with two copies of the risk alleles and had the CC genotype. For the remaining four SNPs, the A allele was non-risk so that those with either the AA or AG genotype were considered low risk and those with the GG genotype were considered high risk.

### Statistical analyses

Statistical Analysis Software v.9.4 (SAS Institute Inc., Cary, NC) was used for all analyses. Subjects with missing information on ethnicity, genotype or biomarkers of interest were excluded. A total of 1,333 subjects were included in the initial analyses, however, 24 subjects did not have genetic information for all five SNPs. These subjects were only included in the analyses where data on the SNPs of interest were available. Genotypes for 9p21 were examined for Hardy-Weinberg equilibrium. Chi-square test was used to assess 9p21 risk allele frequency in different ethnic groups. All continuous variables distributions were tested for normality. Any biological factors that did not fit a normal distribution curve, were log-transformed prior to further analyses however, untransformed means and spreads were reported to facilitate better interpretation of the data.

Principal components analysis (PCA) of food intake scores was used to identify food consumption patterns. These methods have been described in detail elsewhere (Brenner et al., 2011). Briefly, individual food items described in the FFQ were used as the basis of the analyses, and the patterns were obtained through an orthogonal rotation with the varimax rotation function in SAS 9.4. This was done using 0.25 as a loading criterion, with consideration parameters of eigenvalues >1, the scree test, and the qualitative interpretability of patterns. This approach provided a component structure with three independent patterns. Percentage of variance explained by each pattern was not part of the selection criteria. Pattern scores were standardized and normally distributed. The *α*-error was set at 0.05, and *p*-values presented are two-sided. Initially, all analyses were unadjusted, and then adjusted for several covariates. Only those variables that were statistically significant in most models or materially altered the outcomes were retained in the model. The variables in each model were also tested for multicollinearity with tolerance level set at <0.4. No multicollinearity was detected amongst the variables selected for the final models.

Using analysis of covariance (ANCOVA), mean of subject characteristics (i.e., BMI, age *etc.*) was examined across 9p21 genotypes (ie high *versus* low risk groups). In the unadjusted model, multiple linear regression was used to examine if 9p21 genotype, dietary patterns and gene-diet interactions were associated with insulin sensitivity and other biomarkers of CVD among the different 9p21 SNPs. In the final models, analyses were adjusted for age, sex, hormonal contraceptive use, physical activity, and natural log of body mass index. For all analyses, reported *p*-values were 2-sided with statistical significance set at less than 0.05.

## Results

Subject characteristics are summarized in Table 1 in two groups according to rs1333049 homozygous for the risk alleles C (increased risk of CVD) *versus* carriers of non-risk allele G. An additive model was initially assessed, however, the effects were only observed among homozygotes for the risk variant, suggesting a recessive effect. As such, the finalized analyses show the dichotomized groups as carriers of one or no risk alleles *versus* those who are homozygous for the risk allele. Both groups had a similar proportion of Caucasians to East Asians as well as distribution of men and women. Both groups had similar adherence to Prudent, Western and Eastern style dietary patterns, according to the PCA generated scores. G and C allele frequency distributions were also similar among Caucasians and East Asians as well as between men and women.

**TABLE 1:** Subject characteristics according to 9p21 genotype (rs1333049).

	GG + CG (n = 1,035)	CC (n = 298)	*p*
Age (years)	22.7 ± 0.1	22.6 ± 0.1	0.19
Body mass index (kg/m^2^)	22.8 ± 0.1	22.5 ± 0.2	0.11
Waist circumference* (cm)	73.9 ± 0.3	73.9 ± 0.5	0.93
Systolic Blood Pressure (mmHg)	113.9 ± 0.4	114.1 ± 0.7	0.83
Diastolic Blood Pressure (mmHg)	69.2 ± 0.2	69.6 ± 0.5	0.40
Prudent Diet Score	2.98 ± 0.05	2.94 ± 0.09	0.38
Eastern Diet Score	1.23 ± 0.03	1.22 ± 0.05	0.27
Western Diet Score	1.15 ± 0.03	1.12 ± 0.04	0.52
Females (%)	70	66	0.31
Males (%)	30	34	0.22
Caucasian (%)	57	59	0.59
East Asian (%)	43	41	0.58

Values are mean ± standard error, *p*-values are for comparison between three genotypes using unadjusted linear regression model. *Variables were log-transformed to normalize distribution for model building. CRP, C-reactive protein; HDL, high-density lipoproteins; LDL, low density lipoproteins; TG, triglycerides.

All five SNPs were in high linkage disequilibrium (LD) (>80%). When traditional biomarkers of CVD risk were analyzed according to rs1333049 genotype, fasting insulin was the only outcome that was significantly different between the risk *versus* non-risk groups (*p* = 0.04). No other associations were observed between the subgroups or with any other biomarkers (Table 2).

**TABLE 2:** Biomarkers of CVD Risk by 9p21 Genotype (rs1333049) and ethnic group

Subgroup		Genotype		
	CVD biomarker	GG + GC	CC	*p*
All (n = 1,333)	Glucose (mmol/L)	4.77 ± 0.01	4.77 ± 0.02	0.78
	Insulin* (pmol/L)	43.7 ± 0.9	46.8 ± 2.1	0.04
	CRP* (mmol/L)	1.17 ± 0.08	1.18 ± 0.14	0.68
	TG* (mmol/L)	0.97 ± 0.02	0.98 ± 0.03	0.93
	HDL* (mmol/L)	1.56 ± 0.01	1.55 ± 0.02	0.79
	LDL (mmol/L)	2.27 ± 0.02	2.24 ± 0.04	0.39
Caucasians (*n* = 773)				
	Glucose (mmol/L)	4.76 ± 0.01	4.74 ± 0.03	0.51
	Insulin* (pmol/L)	44.1 ± 1.5	46.7 ± 3.0	0.16
	CRP* (mmol/L)	1.45 ± 0.11	1.42 ± 0.18	0.73
	TG* (mmol/L)	0.98 ± 0.02	0.98 ± 0.05	0.35
	HDL* (mmol/L)	1.55 ± 0.02	1.54 ± 0.03	0.98
	LDL (mmol/L)	2.27 ± 0.03	2.19 ± 0.05	0.13
East Asians (*n* = 560)				
	Glucose (mmol/L)	4.78 ± 0.02	4.83 ± 0.04	0.24
	Insulin* (pmol/L)	43.1 ± 1.2	46.9 ± 2.9	0.22
	CRP* (mmol/L)	0.79 ± 0.1	0.82 ± 0.21	0.55
	TG* (mmol/L)	0.96 ± 0.04	0.99 ± 0.04	0.20
	HDL* (mmol/L)	1.57 ± 0.02	1.58 ± 0.03	0.95
	LDL (mmol/L)	2.27 ± 0.03	2.31 ± 0.05	0.62
Females (*n* = 920)				
	Glucose (mmol/L)	4.72 ± 0.01	4.71 ± 0.02	0.83
	Insulin* (pmol/L)	45.2 ± 1.3	50.8 ± 2.9	0.06
	CRP* (mmol/L)	1.25 ± 0.09	1.3 ± 0.18	0.75
	TG* (mmol/L)	0.95 ± 0.02	0.96 ± 0.04	0.81
	HDL* (mmol/L)	1.66 ± 0.01	1.66 ± 0.03	0.82
	LDL (mmol/L)	2.26 ± 0.02	2.26 ± 0.04	0.94
Males (*n* = 413)				
	Glucose (mmol/L)	4.89 ± 0.02	4.9 ± 0.04	0.77
	Insulin* (pmol/L)	39.9 ± 1.3	38.9 ± 2.4	0.53
	CRP* (mmol/L)	0.98 ± 0.14	0.94 ± 0.21	0.93
	TG* (mmol/L)	1.03 ± 0.04	1.02 ± 0.06	0.87
	HDL* (mmol/L)	1.33 ± 0.02	1.34 ± 0.03	0.63
	LDL (mmol/L)	2.3 ± 0.04	2.19 ± 0.06	0.17

Values are mean ± standard error, p-values are for comparison between three genotypes using unadjusted linear regression model. *Variables were log-transformed to normalize distribution for model building. CRP, C-reactive protein; HDL, high-density lipoproteins; LDL, low density lipoproteins; TG, triglycerides.

**FIGURE 1:**
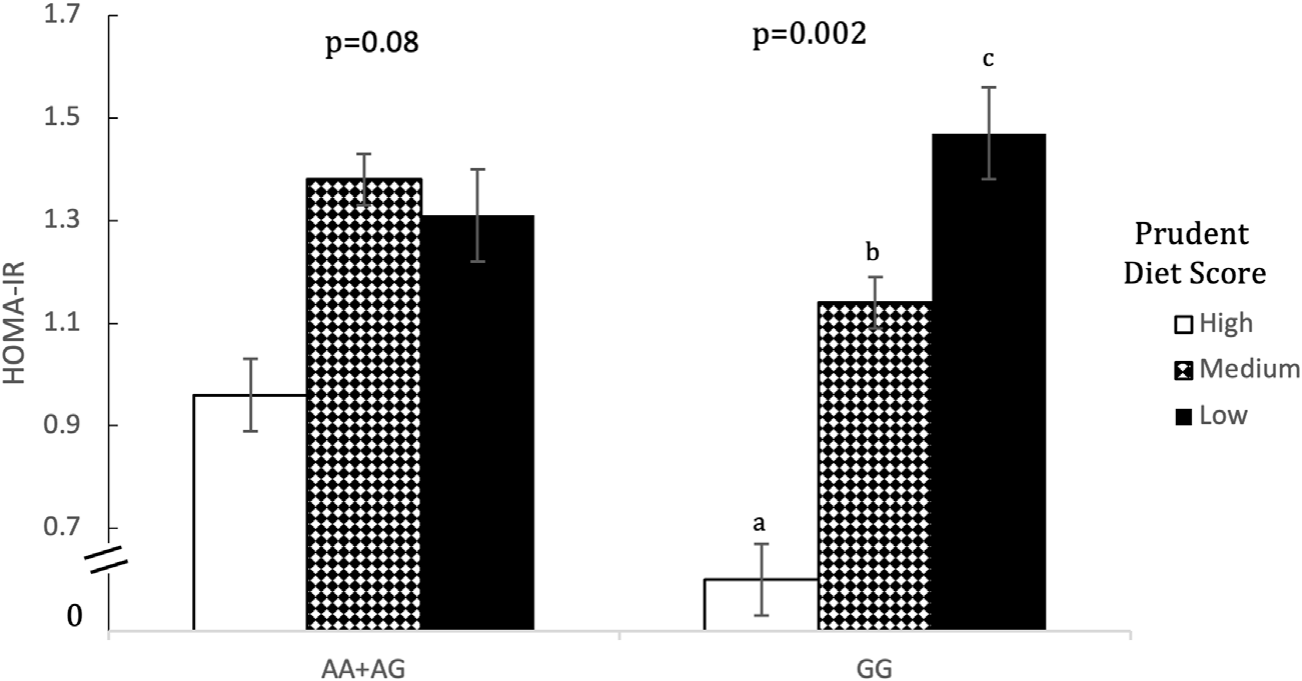
Homeostatic model assessment of insulin resistant (HOMA-IR) by Prudent Dietary Score and 9p21 genotype (rs10757274). Data are means ± SEM. Statistical significance was calculated using unadjusted ANOVA initially for the interaction term of gene-diet in all six groups (*n* = 1309, *p* = 0.031), and then separately for prudent dietary score in those with the AA or AG genotype (*n* = 985, *p* = 0.076) and those with the GG genotype separately (*n* = 324, *p* = 0.002). In the GG group, those with high (75th percentile) and medium (26th-74th percentile) prudent dietary score had a significantly lower HOMA-IR than those with low (25th percentile) prudent diet score (*p* = 0.004 and *p* = 0.014 respectively). In the AA+ AG group HOMA-IR did not differ between any of the prudent dietary score groups. Different letters (a *versus* b) indicate pairwise differences between group means that were statistically different.

**FIGURE 2:**
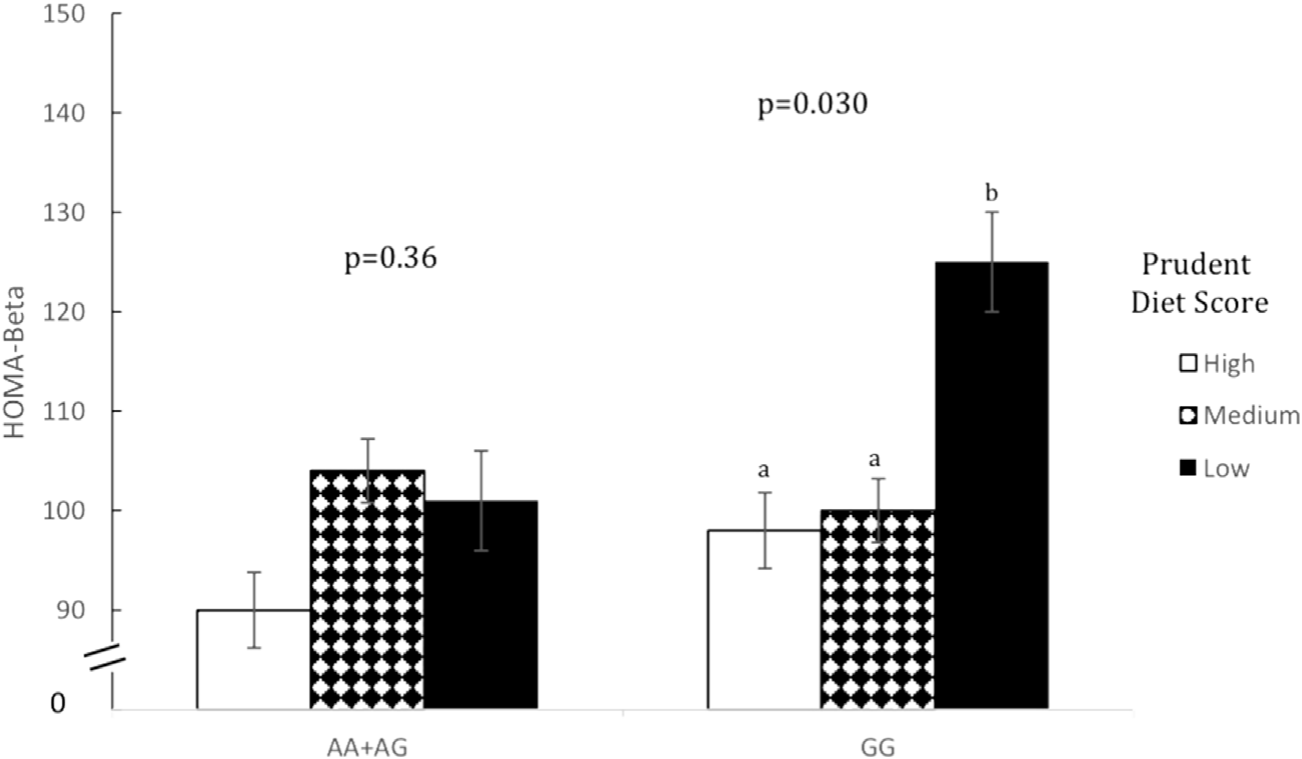
Homeostatic model assessment of beta cell dysfunction (HOMA-Beta) by prudent dietary score and 9p21 genotype (rs10757274).Data are means ± SEM. Statistical significance was calculated using unadjusted ANOVA initially for the interaction term of gene-diet in all six groups (*n* = 1309, *p* = 0.044), and then separately for prudent dietary score in those with AA+ AG genotype (*n* = 985, *p* = 0.360) and those with GG genotype separately (*n* = 324, *p* = 0.030). In the GG group, those with high (75th percentile) and medium (26th-74th percentile) prudent dietary score had a significantly lower HOMA-Beta than those with low (25th percentile) prudent diet score (*p* = 0.013 and *p* = 0.023 respectively). In the AA+ AG group HOMA-Beta did not differ between any of the prudent dietary score groups (*p* = 0.080 and *p* = 0.235, respectively). Different letters (a *versus* b) indicate pairwise differences between group means that were statistically different while same letters indicate pairwise comparison were not statistically different.

Prudent dietary pattern was only significantly associated with fasting insulin, when subjects were stratified according to genotype, for all five SNPs (*p* < 0.001). Western and Eastern dietary patterns showed no significant associations (*p* > 0.05 for all associations). Among those with the high-risk genotype, fasting insulin was on average 1.4 times higher for those consuming a low-prudent diet compared to those consuming a high-prudent diet (*p* < 0.05, Table 3). This association was most evident with rs2383206 (Figure 1), where a Prudent dietary score was inversely associated with HOMA-IR in the GG group (high-risk) (*p* = 0.002), but not in the GA or AA groups (non-risk) (*p* = 0.08). These dietary associations with fasting insulin were not observed in those carrying non-risk alleles. Significant gene-diet interactions on insulin sensitivity using HOMA-IR were observed with all five SNPs, which remained significant after adjusting for covariates (age, sex, hormonal contraceptive use in females, physical activity and log of body mass index) (*p* < 0.05, Table 4). Similar findings were observed when beta-cell dysfunction was estimated *via* HOMA-Beta (Figure 2), however, the association was only significant for rs1333049 (*p* = 0.04) while the other four SNPs did not reach statistical significance (*p* = 0.06 to *p* = 0.10). Adjusting for co-variates did not meaningfully change these associations with HOMA-Beta. In addition to dietary patterns, some food groups were also analyzed for interactions with genotype. Raw vegetables significantly interacted with two 9p21 SNPs (rs10757272 and rs4977574) on HOMA-IR (*p* = 0.025 and *p* = 0.022, respectively, Table 4). Other food groups did not show any significant interactions with any of the SNPs on insulin related outcomes.

**TABLE 3:** Fasting insulin by 9p21 genotype, and prudent dietary score.

	Prudent Dietary Score			*p*-value		
Genotype	Low	Medium	High	Diet	Gene	Interaction
rs1333049				<0.01	0.004	0.01
GC + GG	44.5 ± 23.4	44.8 ± 36.4	39.9 ± 25.0			
CC	58.5 ± 47.0	42.3 ± 24.1	44.7 ± 44.3			
rs10757278				<0.01	0.002	0.02
AA+ AG	44.6 ± 23.4	44.6 ± 36.4	39.8 ± 24.9			
GG	58.8 ± 47.5	42.8 ± 24.3	45.3 ± 45.1			
rs2383206				<0.01	0.039	0.03
AA+ AG	44.4 ± 23.1	44.1 ± 36.6	40.9 ± 30.4			
GG	57.3 ± 45.3	44.5 ± 24.8	41.0 ± 29.8			
rs10757274				<0.01	0.136	0.04
AA+ AG	45.2 ± 28.1	44.4 ± 36.4	41.3 ± 30.6			
GG	56.7 ± 37.3	43.8 ± 24.6	39.5 ± 29.3			
rs4977574				<0.01	0.153	0.05
AA+ AG	45.0 ± 28.0	44.3 ± 36.4	41.4 ± 30.6			
GG	56.4 ± 37.5	43.7 ± 24.6	39.5 ± 29.3			

*Values are mean fasting insulin ±standard error listed in order of increasing prudent dietary score; p-values are for linear regression models with log-fasting insulin as the dependent variable and prudent dietary pattern and binary genotype as the main determinants of interest as well as diet-gene as the interaction term. All models were adjusted for: sex, hormonal contraceptives in females, physical activity, log-body mass index, log-waist circumference. In all SNPs an A>G substitution was noted as the risk allele, however for rs1333049 a G>C substitution signified risk allele as C, therefore the order for the genotype of this SNP is listed as GG, GC, CC.*

**TABLE 4:** Interaction *p*-values Between Dietary Components and 9p21 SNPs on HOMA-IR.

	Dietary patterns			Food groups									
9p21 SNP	Prudent	Western	Eastern	Fresh fruits	Raw vegetables	Cooked vegetables	Whole grains	White grains	Other meat	Processed meat	Total dairy	Total yogurt	Sweets
rs1333049	0.031	0.239	0.716	0.890	0.239	0.263	0.258	0.796	0.765	0.068	0.396	0.066	0.716
rs2383206	0.007	0.141	0.999	0.476	0.109	0.172	0.371	0.484	0.701	0.145	0.550	0.329	0.337
rs10757278	0.009	0.253	0.999	0.759	0.234	0.275	0.244	0.799	0.573	0.081	0.396	0.162	0.655
rs10757274	0.035	0.397	0.958	0.410	0.025	0.340	0.279	0.680	0.822	0.143	0.254	0.053	0.259
rs4977574	0.038	0.279	0.968	0.354	0.022	0.319	0.667	0.740	0.844	0.198	0.264	0.144	0.310

Distribution of dietary components and SNPs were assessed for normality and log-transformed if significantly deviated from a normal distribution. Interaction tests were performed using general linear regression adjusted for main effects of SNP and dietary component. Significance was set at *p*-value of <0.05 and minimum sample size included for analyses was n = 1,309.

## Discussion

Variations in 9p21 are the most robust genetic markers of CVD outcomes in genome wide association studies to date, however, few studies have assessed if gene-environment interactions modify these risks, particularly in young adults. Since environmental factors play an important role in determining CVD risk, study designs that analyze both modifiable and non-modifiable risk factors may be more informative than each approach alone. Furthermore, advanced age is a major player in accumulative effect of multiple factors that lead to development of CVD, studying a young-adult cohort eliminates such confounders. Therefore, we sought to assess the interactions between three common dietary patterns with the five most widely studied 9p21 SNPs on insulin sensitivity and beta-cell dysfunction outcomes in over 1,300 young-adults representing two different ethnic groups. To our knowledge, this study is the first to examine these associations in a cohort of young adults.

We found that those with the 9p21 risk genotype had higher fasting plasma insulin than the non-risk group. In addition, HOMA-IR and HOMA-Beta were also elevated among those homozygous for the risk alleles, particularly in those consuming a low prudent diet. IR earlier in life is often predictive of CVD in middle and late adulthood, even in those without diabetes (Gast et al., 2012). In addition to traditional pathways, IR has also been linked to atherosclerosis through pro-inflammatory activity on vascular and immune cells (Bertoni et al., 2007; Montecucco et al., 2008). Mechanisms by which 9p21 risk alleles are associated with CVD are largely unknown, however, there is some evidence that endothelial dysfunction might be one of the pathways (Harismendy et al., 2011). The same mechanisms that contribute to endothelial dysfunction such as interferon-γ activation, have also been shown to contribute to IR in human adipose tissue (McGillicuddy et al., 2009). Therefore, our findings, are in agreement with these observations and might aid in further understanding of the role of 9p21 in CVD risk. It is important to note that none of the components of the metabolic syndrome including fasting glucose, HDL, triglyceride, waist circumference or systolic blood pressure were associated with the risk genotype or elevated CRP levels. These further allude to the possibility of elevated fasting insulin in isolation initiating the cascade of metabolic syndrome and other possible CVD mechanisms that have been associated with the risk genotype in other studies. Given the young age of our cohort (mean age 22.6) this could further explain the lack of association of 9p21 risk factors with typical CVD or metabolic syndrome markers. Moreover, studying a young adult population enables us to identify early metabolic changes associated with 9p21 risk variants.

Mean HOMA-IR was 150% higher and HOMA-Beta was 30% higher in those with a low Prudent dietary pattern score *versus* those with a high Prudent dietary pattern from the same high-genetic risk group (*p* = 0.004, *p* = 0.013, respectively). Higher Prudent dietary pattern scores appeared protective against IR and beta-cell dysfunction in the risk group and this protection was in a stepwise fashion with HOMA-IR. Meanwhile in the non-risk group, Prudent dietary patterns were not associated with HOMA-IR or HOMA-Beta. Although associations between nutrition, fasting insulin and 9p21 have not been previously reported, others have reported on 9p21 gene-diet interactions with related outcomes including glycemic control (Doria et al., 2008; Hindy et al., 2014), lipid profile (Mehramiz et al., 2018) and CVD events (Do et al., 2011; Hindy et al., 2014). Our findings are in agreement with these previous findings and add to the evidence that CVD risk factors may be modifiable in young adults with high risk 9p21 genotypes. It is also important to note that these findings could explain previous inconsistencies linking one-size-fits-all dietary interventions to CVD outcomes (Jenkins et al., 2017) and might warrant a more personalized approach to dietary interventions to prevent CVD (Garcia-Bailo and El-Sohemy, 2021). We also acknowledge that replication of these analyses in other cohorts would add to the body of evidence.

We have demonstrated higher Prudent pattern scores in the current study had strong correlations with increased vitamin and mineral levels (vitamins A, B1, B2, B6, C, D, and K, magnesium, and iron) and this was likely due to the relatively large presence of fruits and vegetables in this dietary pattern (Brenner et al., 2011). Higher intake of fruits and vegetables have been shown to be protective towards CVD risks in several dietary patterns and populations (Alissa and Ferns, 2017). Several mechanisms have been proposed by which these foods may be protective and include; *via* bioactive components with antioxidant, anti-inflammatory, and electrolyte properties, as well as functional properties, such as low glycemic load and low energy density (Alissa and Ferns, 2017). In the current study, high fruits and vegetable intake was a distinguishing feature of both the Prudent dietary patterns and the Eastern dietary patterns, however, no associations were found with the Eastern dietary patterns and 9p21 genotype on insulin sensitivity. One of the reasons for this lack of association with the Eastern pattern might be that the majority of fruits and vegetables in this dietary pattern was cooked or processed whereas the fruits and vegetables in the Prudent dietary patterns were often freshly consumed with minimal processing steps. This association was evident when food groups were analyzed and consumption of fresh vegetables were shown to interact with two 9p21 SNPs on HOMA-IR (*p* = 0.022 and 0.025, Table 4). Our finding is in agreement with two other studies where intake of fresh vegetables was protective against CVD in high-risk genotype in 9p21 (Do et al., 2011; Hindy et al., 2014).

Higher Western dietary patterns in the current population were associated with high sodium, fat, saturated fat and energy intake (Brenner et al., 2011), likely because of the high proportion of processed foods included in this dietary pattern. Interestingly, this dietary pattern was not associated with insulin markers and 9p21 genotype. This finding is also in agreement with another study that found while Prudent dietary patterns attenuate the 9p21 genetic risk, Western dietary patterns did not affect CVD events (Do et al., 2011). This is contrary to other findings where Western dietary patterns have been linked to CVD and diabetes among many other chronic conditions (McCullough et al., 2002; Carrera-Bastos et al., 2011). Although one large systematic review found strong evidence to support a causal relationship between Prudent/Mediterranean dietary factors and reduction in CVD, they concluded that there was insufficient evidence against components of a Western dietary pattern (including saturated fats) to support a causal relationship with increased CVD (Mente et al., 2009). In the same systematic review, however, there was evidence for trans-fats being a contributing factor towards CVD and western diets are typically high in trans-fats (Mente et al., 2009). In the current study, however, our dietary patterns did not characterize trans-fats as major components of any of the three dietary patterns (Brenner et al., 2011).

Linear regression model. Chi-square test was used to test for differences between genotypes in categorical variables. *Variables were log-transformed to normalize distribution before use in regression.

## Data Availability

The authors acknowledge that the data presented in this study must be deposited and made publicly available in an acceptable repository, prior to publication. Frontiers cannot accept a manuscript that does not adhere to our open data policies.

## Abbreviations

SNP; single nucleotide polymorphism, TG; triglyceride, IR; insulin resistance TNHS; Toronto Nutrigenomics and Health Study, ED; endothelial dysfunction, DM2; type 2 diabetes, HOMA-IR; Homeostatic function, PCA; principal components analysis, FFQ; food frequency questionnaire, LD; linkage disequilibrium.
